# CORO6 Promotes Cell Growth and Invasion of Clear Cell Renal Cell Carcinoma via Activation of WNT Signaling

**DOI:** 10.3389/fcell.2021.647301

**Published:** 2021-05-07

**Authors:** Xinjun Wang, Yiming Xiao, Si Li, Zhijian Yan, Guangcheng Luo

**Affiliations:** ^1^Department of Urology, Zhongshan Hospital Xiamen University, Xiamen, China; ^2^The School of Clinical Medicine, Fujian Medical University, Fujian, China

**Keywords:** CORO6, rcc, prognosis, Wnt signaling, invasion

## Abstract

Renal cell carcinoma (RCC) constitutes the most lethal type of genitourinary cancer. Understanding of RCC tumor biology helps to identify novel targets and develop directed treatments for patients with this type of cancer. Analysis from both The Cancer Genome Atlas Kidney Renal Clear Cell Carcinoma dataset and our RCC samples demonstrated that the expression level of CORO6 was significantly higher in RCC patients than in normal kidney tissues, and its level was highly associated with tumor stage and grade. Importantly, CORO6 expression level was an independent predictor of tumor metastasis and overall survival in RCC patients. Our cell line data also confirmed that CORO6 knockdown could suppress RCC cell growth as well as cell migration and invasion. The depletion of CORO6 led to cell cycle arrest at the G0/G1 phase and caused cell apoptosis. Further, mechanistic dissection showed that CORO6 mediated RCC cell growth, and cell invasion relied on WNT signaling. Moreover, the *in vivo* data suggested that CORO6 knockdown indeed suppressed RCC tumor growth. Overall, our study defines the oncogenic role of CORO6 in RCC progression and provides a rationale for developing CORO6-targeted therapies for improved treatment of RCC patients.

## Introduction

Renal cell carcinoma (RCC) is the most common type of kidney cancer, and its incidence continues to rise (Siegel et al., 2019). Clear cell renal cell carcinoma (ccRCC) is the most common type of RCC and accounts for ~80% of all cases (Choueiri et al., 2007; Yin et al., 2020). Understanding of the biological pathogenesis of ccRCC has led us to identify that von Hippel-Lindau tumor suppressor (VHL)/hypoxia-inducible factor 2 alpha (HIF2α signaling plays a central role in its development. VHL inactivation stabilizes HIF2α, which in turn drives hypoxia-induced gene expression, such as vascular endothelial growth factor (VEGF) (Ratcliffe, 2003; Mcronald et al., 2009; Banumathy and Cairns, 2010; Zhai et al., 2017). VEGF induction promotes new blood vessel formation, thereby providing nutrients for tumor cells (Kaelin, 2002; Choueiri, 2011). Therefore, anti-angiogenesis reflects one of the treatment options for ccRCC patients. Sunitinib, sorafenib, and pazopanib are all anti-angiogenic drugs that significantly benefit ccRCC patients (Lang and Harrison, 2010; Choueiri, 2011; Keisner and Shah, 2011; Kilonzo et al., 2013). However, patients will eventually progress to the metastatic stage, which only has an ~10% likelihood of 5-year survival. Therefore, novel and effective therapies are urgently needed for improved treatment of metastatic RCC patients.

CORO6 (Coronin 6 or Coronin-Like Protein E) is a member of the coronin family of proteins with a conserved WD40 repeat domain (Chan et al., 2011; Ren et al., 2012). By interacting with F-actin and Arp2/3, coronin family members have the capacity to inhibit actin dynamics. Recent studies have demonstrated that CORO proteins were significantly increased in metastatic cancers, such as glioma, lymphoma, RCC, gastric cancer, and hepatocellular carcinoma (Thal et al., 2008; Luan et al., 2010; Wu et al., 2010; Ren et al., 2012). For instance, CORO3 depletion could suppress gastric cancer metastasis by reducing the expression levels of matrix metallopeptidase 9 (MMP9) and cathepsin K (Ren et al., 2012). Similar to other coronin members, one early report suggested that CORO6 was over-induced in breast cancer, suggesting that it may also play an oncogenic role in cancer development. However, the role of CORO6 in RCC progression has not yet been investigated.

The WNT signaling pathway is highly involved in cancer development, including RCC (Zhan et al., 2017). Generally, binding of WNT to the Frizzled (Fzd) receptor leads to the phosphorylation of Disheveled (Dvl), which in turn inhibits glycogen synthase kinase (GSK-3β) activity. The inactivation of GSK-3β leads to the dephosphorylation and stabilization of β-catenin, which enters the nucleus and regulates a gene expression spectrum with the help of lymphoid enhancer-binding factor (LEF) and T-cell factor (TCF) (Bafico et al., 1999; Polakis, 2000). C-myc and CCND1 are two classical downstream genes of β-catenin, suggesting that WNT/β-catenin signaling is highly involved in the regulation of cancer growth and metastasis (Rohrs et al., 2009).

In this study, we analyzed The Cancer Genome Atlas Kidney Renal Clear Cell Carcinoma (TCGA KIRC) dataset and found that CORO6 was remarkably increased in ccRCC patients compared to normal kidney tissues. The CORO6 expression level was highly associated with tumor stage, tumor grade, tumor metastasis, and overall survival. All of these analyses suggest that CORO6 may causally contribute to ccRCC development. Indeed, our experimental results revealed that CORO6 knockdown could suppress cell growth as well as cell migration and invasion of RCC cells, supporting the oncogenic role of CORO6 in RCC development. Mechanistic dissection demonstrated that WNT signaling activation was at least one of the mechanisms responsible for CORO6-induced RCC cell growth and invasion. Importantly, the *in vivo* data also confirmed that CORO6 depletion suppressed RCC tumor growth. Together, our data define the tumor-promoting role of CORO6 in ccRCC progression and provide a compelling rationale to develop CORO6-targeted therapies for improved treatment of ccRCC patients.

## Materials and Methods

### Patient Samples

CcRCC samples (40) and their paired normal tissues (40) were obtained from the Department of Urology, Zhongshan Hospital Xiamen University (Xiamen, China). Resected tissues were stored in liquid nitrogen for further use. Informed consent was obtained from all patients, and the study was approved by the Institutional Review Board of Xiamen University.

### Cell Culture

Caki-1, SN12-PM6, and 293T cells were purchased from the American Type Culture Collection (Manassas, VA, USA). The cells were maintained in 10% fetal bovine serum (FBS) with Dulbecco's Modified Eagle Medium (DMEM; 100 units/mL penicillin and 100 μg/mL streptomycin) and cultured in a humidified 5% CO_2_ environment at 37°C.

### Lentivirus Generation

Plasmids (pLKO-based or pWPI-based) were co-transfected with psPAX2 and pMD2.G into 293T cells using the standard calcium phosphate transfection method as previously reported. After 48 h, the supernatant was collected using a 0.45 μm filter and infected RCC cells in the presence of 8 μg/mL polybrene. shCORO6 was cloned into PLKO while PWPI was lentiviral backbone for CORO6 cDNA. shRNA infected RCC cells were selected with 1 μg/ml puromycin.

### Western Blotting

Caki-1 and SN12-PM6 cells were lysed in cold radioimmunoprecipitation assay (RIPA) lysis buffer, and the lysates were loaded onto 10–12% sodium dodecyl sulfate polyacrylamide gel for electrophoresis. Separated proteins were transferred to polyvinylidene difluoride (PVDF) membranes and probed with specific primary antibody (1:1,000 dilution) overnight at 4°C followed by incubation with 1:10,000 HRP-conjugated secondary antibody for 1 h. The blots were analyzed using enhanced chemiluminescence plus reagents and scanned with the Storm Electrophoresis Scanner (Amersham Pharmacia Biotech Inc., Piscataway, NJ, USA). The antibodies used in this study were as follows: c-Myc (10828-1-AP; Proteintech, Rosemont, IL, USA), CORO6 (17243-1-AP; Proteintech), CCND1 (60186-1-lg; Proteintech), GAPDH (60004-lg; Proteintech), and AXIN2 (ab109307; Abcam, Cambridge, UK).

### Real-Time Quantitative Reverse Transcription Polymerase Chain Reaction (qRT-PCR)

Total RNA was isolated using Trizol reagent (Invitrogen, Carlsbad, CA, USA). Next, 1 μg of total RNA was subjected to reverse transcription using Superscript III transcriptase (Invitrogen). Real-time qRT-PCR was performed using the Bio-Rad CFX96 system (Bio-Rad Laboratories, Hercules, CA, USA) with SYBR Green Master Mix to determine the mRNA expression levels of target genes. The expression levels were normalized to the 18s rRNA level.

### 3-(4,5-Dimethylthiazolyl)-2,5-diphenyltetrazolium Bromide (MTT) Cell Growth Assay

Caki-1 and SN12-PM6 cells with or without CORO6 manipulation were seeded into 24-well-plates at a density of 3,000 cells/well. The cells at different time points (Day 0, Day 1, Day 2, and Day 3) were incubated with 5 μg/mL MTT (Sigma-Aldrich, St. Louis, MO, USA) for 2 h. Then, 500 μL dimethyl sulfoxide (DMSO) was added to each well, and the absorbance was read at 490 nm.

### The EdU Proliferation Assay

It was performed according to the manufacturer's instructions (RiboBio, Guangzhou, China). After transfection for 24 h, Caki-1 and SN12-PM6 cells were cultured in DMEM containing EdU at 37°C for 6 h. Then, cells were fixed with 4% formaldehyde for 20 min, followed by treatment with glycine for 5 min. We used treatment with 0.5% Triton X-100 at 28°C for 10 min to permeabilize the cell membranes. After washing twice, each well was treated with 200 μL of 1x Apollo reaction cocktail for 20 min. Subsequently, nuclear DNA was stained with Hoechst and imaged using fluorescence-based microscopy (Motic, Hongkong, China).

### Flow Cytometry

Caki-1 and SN12-PM6 cells with or without CORO6 manipulation were collected and fixed with 75% ethanol. The cells were stained with either propidium iodide (PI; cell cycle analysis) or annexin V (apoptosis) for 10 min and subjected to flow cytometry analysis.

### Wound Healing Assay

Caki-1 and SN12-PM6 cells with or without CORO6 manipulation were scratched using a pipette tip. After 48 h, images of these cells were captured using microscopy.

### Transwell Assay

Approximately 5 × 10^4^ Caki-1 or SN12-PM6 cells with or without CORO6 manipulation were loaded into the upper chamber of the inserts, which were pre-coated with 1:5 diluted Matrigel (BD Biosciences, Franklin Lakes, NJ, USA). A medium containing 0% FBS in the lower chamber served as a chemoattractant. After 12 h of incubation, the cells that did not invade through the pores were carefully wiped out with cotton wool. Then, the invading cells in the inserts were stained with crystal violet and imaged with an IX71 inverted microscope (Olympus Corporation, Tokyo, Japan). Experiments performed on inserts without Matrigel were considered for the migration assay.

### *In vivo* Xenografted Mouse Model

Approximately 1 × 10^6^ Caki-1 cells (shCtrl or shCORO6) within 200 μl Matrigel (BD, Inc., Franklin Lakes, NJ) were subcutaneously implanted into nude mice to allow for tumor growth. After 18 days, the tumors were measured using calipers every 4 days. After 38 days post-implantation, the mice were sacrificed, and the tumors were removed for detection of RNAs or proteins.

### Immunohistochemical Staining (IHC)

Tissues were fixed in 10% (v/v) formaldehyde in PBS and embedded in paraffin. Embedded tissues were cut into 5 μm sections and deparaffination was performed. The sections were treated with citrate buffer (pH 6.0) at 98°C for 20 min. The slides were incubated with endogenous peroxidase blocking solution, and then incubated with the CORO6 (17243-1-AP, Proteintech) antibody at 4°C overnight. After being rinsed with PBS, the slides were incubated for 45 min with biotin-conjugated secondary antibody, washed, and then incubated with enzyme conjugate horseradish peroxidase (HRP)-streptavidin. Freshly prepared DAB was used as substrate to detect HRP.

### Kyoto Encyclopedia of Genes and Genomes (KEGG) Pathway Analysis

RNA sequencing from the TCGA-KIRC dataset and the corresponding clinical information were downloaded from the Xena Functional Genomics Explorer (https://xenabrowser.net/heatmap/) of the University of California, Santa Cruz (Santa Cruz, CA, USA). KEGG pathway analysis was performed using the Gene Set Enrichment Analysis (GSEA) online tool (http://www.broadinstitute.org/gsea) after dividing patients into two groups using the median expression level of CORO6.

### Statistical Analysis

All statistical analyses were performed using GraphPad Prism software (GraphPad Software, San Diego, CA, USA). Data are presented as means ± standard errors (SEs). Differences were analyzed with the Student's *t*-test, and the significance level was set at *P* < 0.05. *, **, and *** indicate *P* < 0.05, *P* < 0.01, and *P* < 0.001, respectively.

## Results

### CORO6 Upregulation in ccRCC Patients

**Figure 1:**
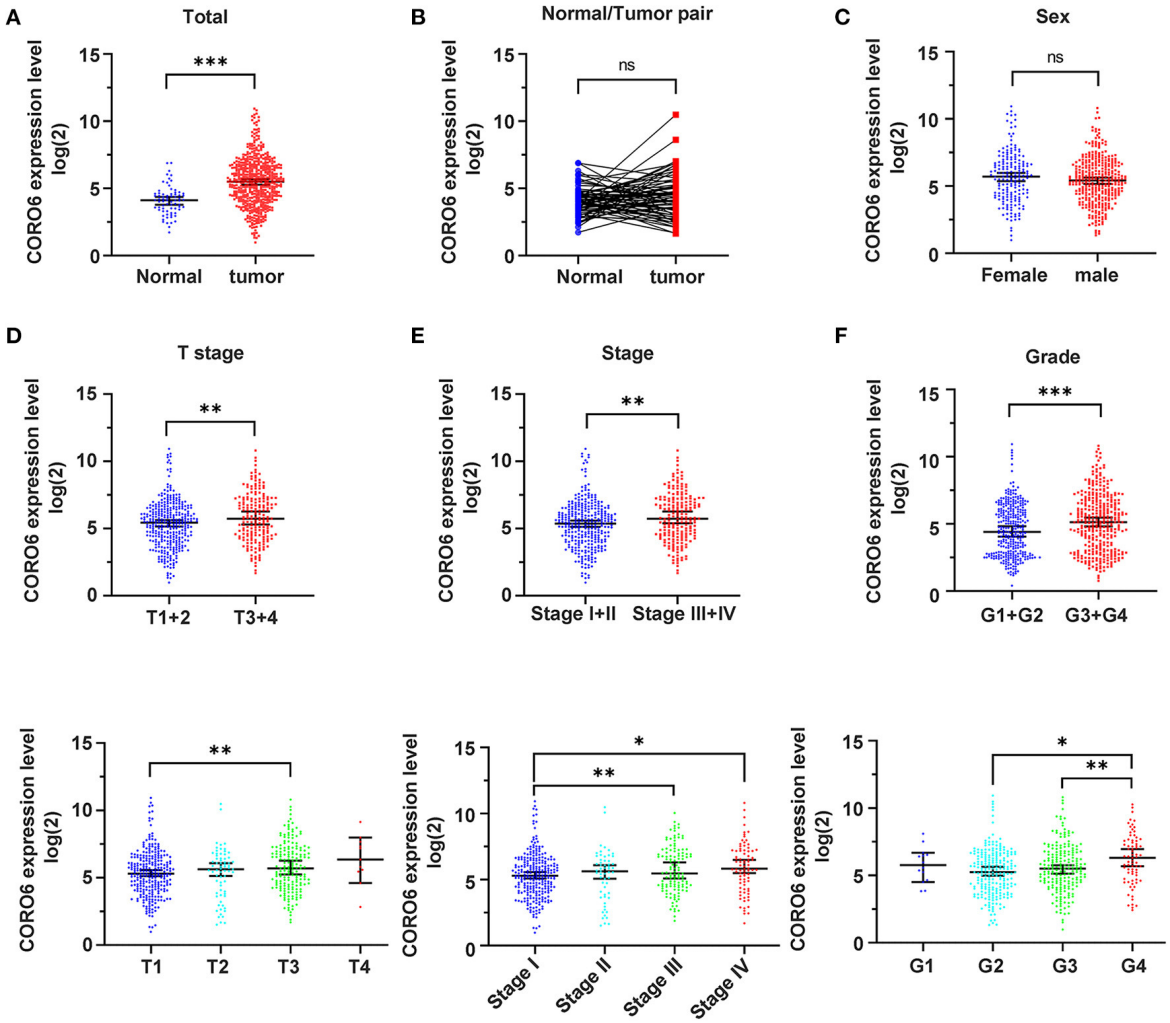
CORO6 upregulation in ccRCC patients. **(A)** The CORO6 mRNA level was significantly increased in ccRCC patients (*n* = 533) compared to normal kidney tissues (*n* = 72). **(B)** The CORO6 mRNA level was indistinguishable between ccRCC (*n* = 72) and paired adjacent tissues (*n* = 72). **(C)** The CORO6 expression level displayed no correlation with sex hormones. **(D)** Top: the CORO6 mRNA level was upregulated in ccRCC patients with T stage III+IV compared to patients with T stage I+II. Bottom: ccRCC patients with T stage III tended to express a higher level of CORO6 compared to patients with T stage I. **(E)** Top: the CORO6 mRNA level was elevated in ccRCC patients with TNM stage III+IV compared to patients with TNM stage I+II. Bottom: ccRCC patients with TNM stage III or IV tended to express a higher level of CORO6 when compared to patients with TNM stage I. **(F)** Top: the CORO6 mRNA level was upregulated in ccRCC patients with G3+G4 compared to patients with G1+G2. Bottom: ccRCC patients with G4 tended to express a higher level of CORO6 compared to G2+G3. One way ANOVA test was performed in bottom panels of **(D–F)**. *P < 0.05, **P < 0.01, and ***P < 0.001. Ns, no significance.

To explore the clinical significance of CORO6, we first examined its mRNA level in ccRCC patients and normal kidney tissues from the TCGA KIRC dataset. Our analysis revealed that the CORO6 mRNA level was clearly increased in ccRCC tissues compared to normal kidney tissues (Figure 1A). However, the CORO6 mRNA level was indiscriminate in 72 pairs of ccRCC samples and their corresponding non-cancerous tissues (Figure 1B). The CORO6 expression level was also not associated with sex hormones (Figure 1C). Interestingly, upregulation of CORO6 was observed in tumor (T) stages III and IV compared to T stages I and II (Figure 1D, top). Moreover, the expression level of CORO6 was also highly associated with grade (G), stage, and pathological tumor/node/metastasis (TNM Classification of Malignant Tumors; Union for International Cancer Control) stage in ccRCC patients (Figures 1E,F, top). Of note, a higher CORO6 level was observed in T stage III, TNM stage III/IV, and G4 compared to the corresponding controls (Figures 1D–F, bottom). Taken together, all of these data suggest that the CORO6 level is significantly upregulated in ccRCC patients and is further increased as the tumor progresses to the lethal stage.

### Association Between CORO6 Level and Poor Prognosis in ccRCC Patients

**Figure 2:**
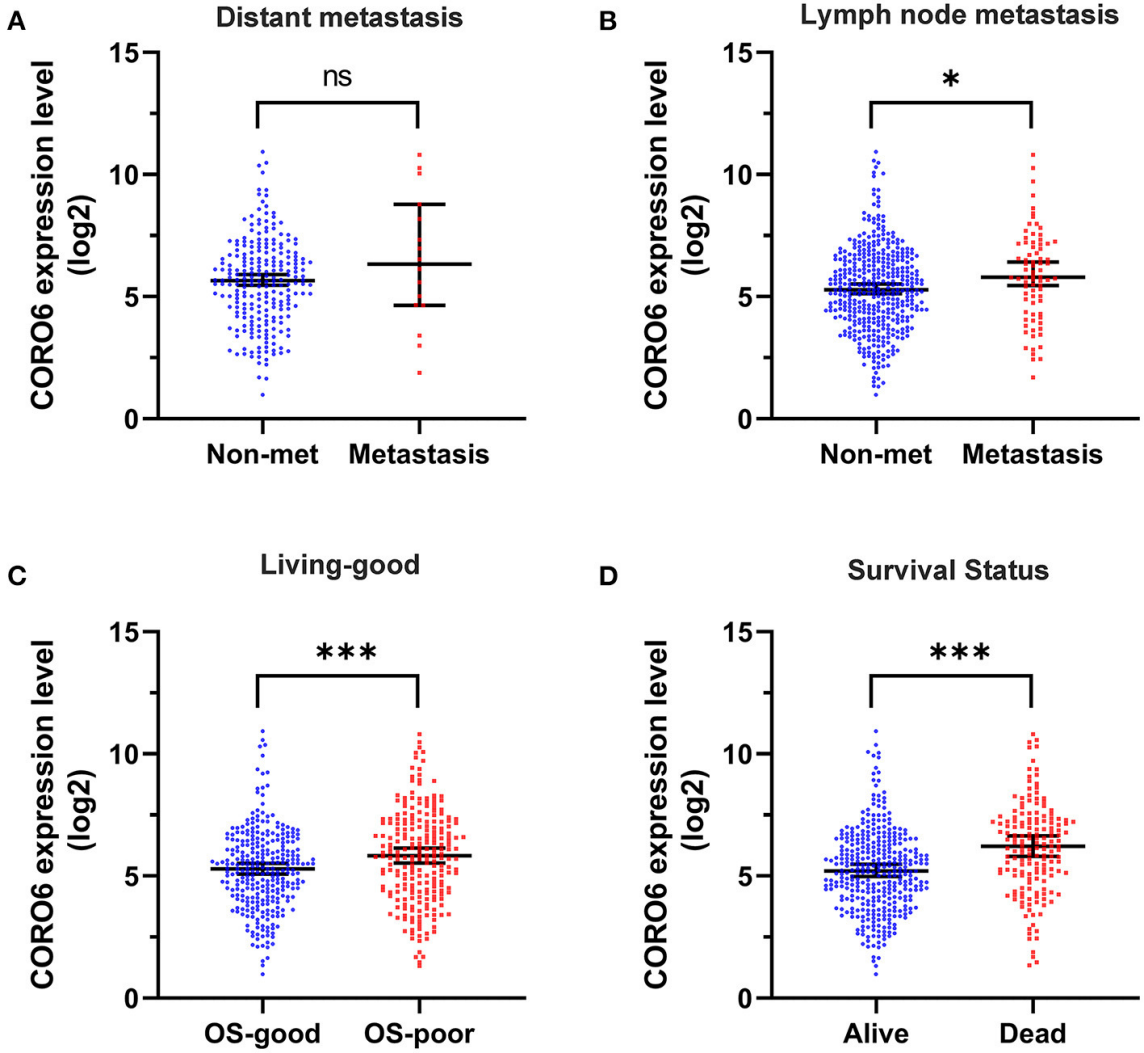
High CORO6 level was an independent predictor of poor prognosis in ccRCC patients. A high expression level of CORO6 is strongly associated with tumor metastasis **(A,B)**, poor OS **(C)**, and death **(D)**. *P < 0.05 and ***P < 0.001. Ns, no significance.

We then sought to investigate whether the expression level of CORO6 was associated with ccRCC progression. Data revealed that ccRCC patients with distant metastasis tended to express a high level of CORO6 compared to non-metastatic patients (Figure 2A), although a statistical significance was not reached owing to the limited sample size of metastatic ccRCC patients in TCGA KIRC dataset. Of note, the CORO6 level was significantly elevated in lymph node metastatic ccRCC patients (Figure 2B). Importantly, the CORO6 mRNA level clearly classified the overall survival (OS) of ccRCC patients (Figure 2C), in which patients with poor OS tended to express a high level of CORO6. Our analysis also showed that CORO6 was highly expressed in dead ccRCC patients compared to living ones (Figure 2D). Together, all of these data support the notion that a high CORO6 level serves as an independent predictor of poor prognosis in ccRCC patients.

### Correlation Between CORO6 Level and OS in ccRCC Patients

**Figure 3:**
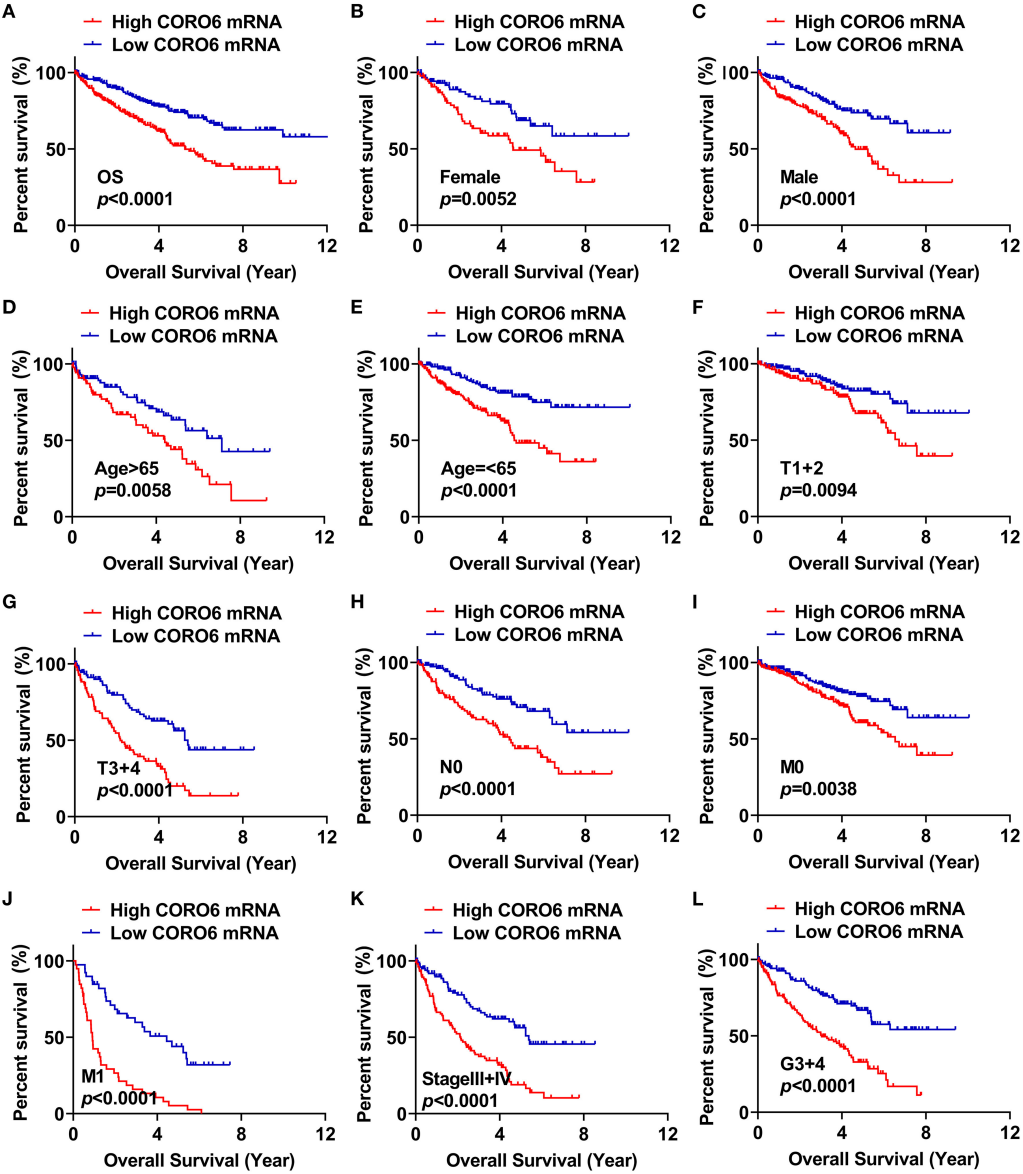
CORO6 level was highly correlated with the OS of ccRCC patients. **(A)** CcRCC patients with a high expression level of CORO6 had a shorter OS time. **(B–L)** OS analyses of CORO6 in ccRCC patients classified by female **(B)**, male **(C)**, age > 65 **(D)**, age ≤ 65 **(E)**, T1+T2 **(F)**, T3+T4 **(G)**, N0 **(H)**, M0 **(J)**, M1 **(K)**, TNM stage III+IV **(M)**, and G3+G4 **(L)**.

The correlation between CORO6 level and the OS of ccRCC patients was further analyzed. We first divided the 533 patients from the TCGA-KIRC dataset into two groups using the median expression level of CORO6 as a cutoff. Figure 3A demonstrates that patients with a high CORO6 level had a shorter OS time (*P* < 0.0001). Furthermore, OS analyses among various subgroups of ccRCC patients were performed. The results demonstrated that CORO6 expression level may serve as a prognostic factor for ccRCC patients with different classifications, such as female (Figure 3B; *P* = 0.0052), male (Figure 3C; *P* < 0.0001), age > 65 years (Figure 3D; *P* = 0.0058), age ≤ 65 years (Figure 3E; *P* < 0.0001), T stage I+II (Figure 3F; *P* = 0.0094), T stage III+IV (Figure 3G; *P* < 0.0001), N0 (Figure 3H; *P* < 0.0001), M0 (Figure 3I; *P* = 0.0036), M1 (Figure 3J; *P* < 0.0001), TNM stage III+V (Figure 3K; *P* < 0.0001), and G3+G4 (Figure 3L; *P* < 0.0001). However, we failed to observe a significant association between CORO6 expression and the OS of ccRCC patients with TNM stage I+II and G1+G2 (data not shown). Collectively, all of these analyses indicate that CORO6 expression level is tightly correlated with the OS of ccRCC patients.

### Experimental Validation of CORO6 Expression in ccRCC Samples and RCC Cell Lines

**Figure 4:**
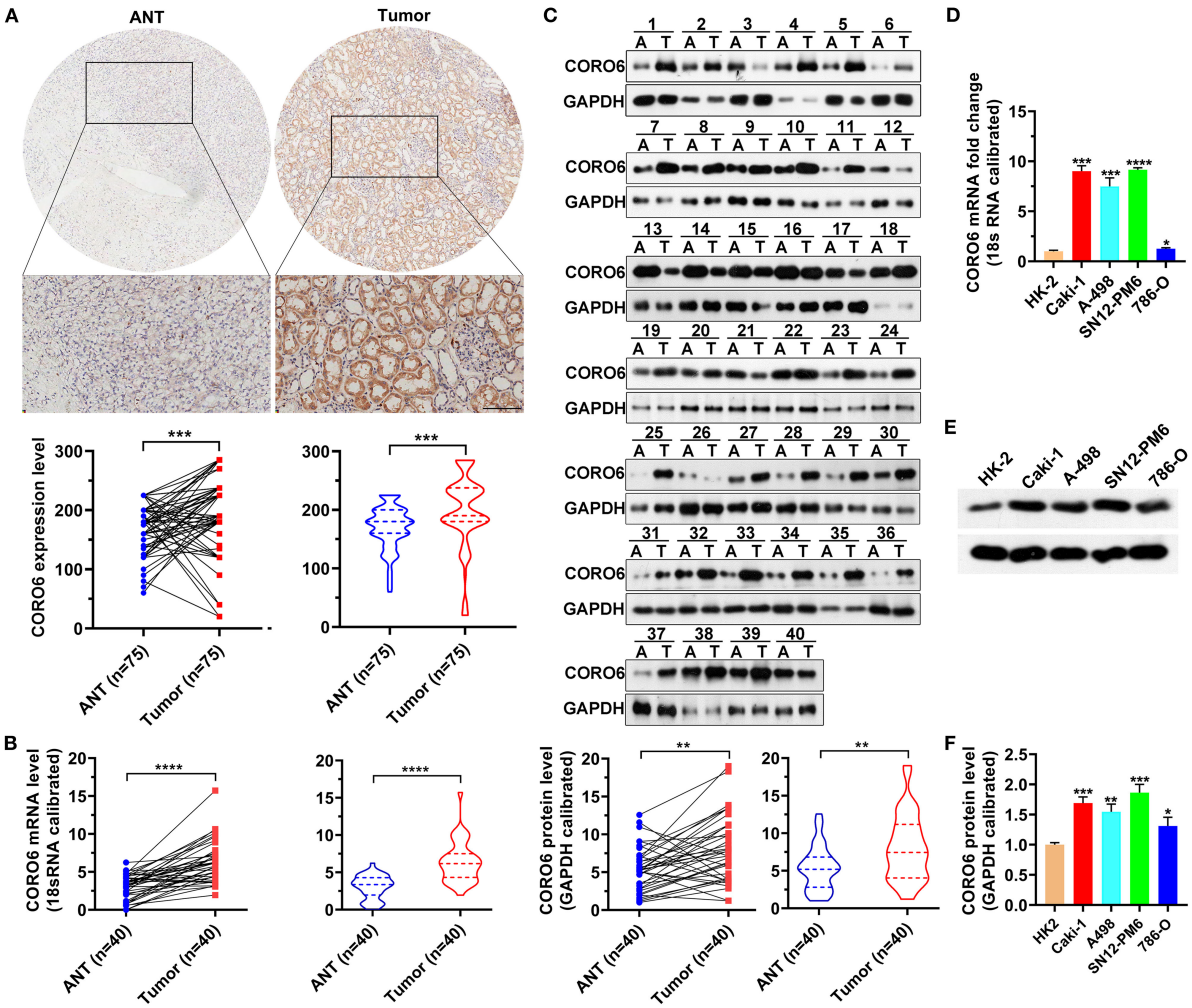
Experimental validation of CORO6 expression in ccRCC samples and RCC cell lines. **(A)** IHC staining showed that CORO6 was overexpressed in ccRCC tumors. Top, representative images of CORO6 IHC staining in tissue microarrays. Bottom, quantification of CORO6 IHC staining in tissue microarrays with ccRCC tumors (*n* = 75) and adjacent normal kidney tissues (*n* = 75). **(B)** The CORO6 mRNA level was considerably increased in our collected ccRCC samples (*n* = 40) compared to paired normal tissues (*n* = 40). 18s rRNA served as the normalizing control. **(C)** The CORO6 protein level was significantly increased in our collected ccRCC samples (*n* = 40) compared to paired normal tissues (*n* = 40). **(C)** Western blotting showed that ccRCC tumors expressed a high level of CORO6. GAPDH served as the internal control. **(D)** The qPCR assay exhibited that RCC cell lines had a higher level of CORO6 compared to normal HK2 cells. Gene expression was normalized to 18s rRNA. **(E)** Western blotting confirmed that the CORO6 protein level was increased in RCC cell lines. GAPDH served as the loading control. **(F)** A quantification of **(E)**. *P < 0.05, **P < 0.01, ***P < 0.001, and ****P < 0.0001. Ns, no significance.

To verify the above online analyses, we applied immunohistochemistry (IHC) to stain CORO6 in tissue microarrays with ccRCC tumors (*n* = 75) and paired adjacent tissues (*n* = 75). The IHC results consistently supported the potential tumor-promoting role of CORO6 in ccRCC development, showing CORO6 was significantly upregulated in ccRCC tumors using the adjacent kidney tissues as controls (Figure 4A, Table 1). We next collected ccRCC samples for the detection of CORO6 at both the protein and mRNA levels. The CORO6 mRNA level was significantly increased in ccRCC patients (*n* = 40) compared to adjacent normal kidney tissues (Figure 4B). Western blotting detection also confirmed that the CORO6 protein level was considerably upregulated in ccRCC samples (Figure 4C). Moreover, RCC cell lines, including Caki-1, A-498, SN12-PM6, and 786-O, tended to highly express CORO6 in contrast to normal kidney cell line HK2 using both quantitative polymerase chain reaction (qPCR; Figure 4D) and western blotting (Figures 4E,F). All these data suggest that CORO6 is highly expressed in RCC cell lines and ccRCC patients and may serve as a tumor-promoting factor in determining ccRCC progression.

**Table 1:** Correlation between clinicopathological variables and CORO6 expression in RCC.

	**Total (*N* = 75)**	**CORO6 Expression**		***P*-value**
		**High**	**Low**	
		**(*N* = 32)**	**(*N* = 43)**	
**Age (year)**				
<65	58 (77.3%)	23 (71.9%)	35 (81.4%)	0.339
≥65	16 (21.3%)	9 (28.1%)	7 (16.3%)	
Unknown	1 (1.3%)	0 (0%)	1 (2.3%)	
**Gender**				
Male	50 (66.7%)	23 (71.9%)	27 (62.8%)	0.54
Female	24 (32.0%)	9 (28.1%)	15 (34.9%)	
Unknown	1 (1.3%)	0 (0%)	1 (2.3%)	
**Pathological grade**				
I	30 (40.0%)	10 (31.2%)	20 (46.5%)	0.365
II	28 (37.3%)	13 (40.6%)	15 (34.9%)	
III	16 (21.3%)	9 (28.1%)	7 (16.3%)	
IV	1 (1.3%)	0 (0%)	1 (2.3%)	
**Clinical stage**				
Phase 1	35 (46.7%)	14 (43.8%)	21 (48.8%)	0.0669
Phase 2	18 (24.0%)	4 (12.5%)	14 (32.6%)	
Phase 3	16 (21.3%)	10 (31.2%)	6 (14.0%)	
Phase 4	6 (8.0%)	4 (12.5%)	2 (4.7%)	
**Pathologic T**				
T1	36 (48.0%)	14 (43.8%)	22 (51.2%)	0.0389
T2	18 (24.0%)	4 (12.5%)	14 (32.6%)	
T3	17 (22.7%)	11 (34.4%)	6 (14.0%)	
T4	4 (5.3%)	3 (9.4%)	1 (2.3%)	
**Pathologic N**				
N0	75 (100%)	32 (100%)	43 (100%)	0.204
**Pathologic M**				
M0	73 (97.3%)	31 (96.9%)	42 (97.7%)	1
M1	2 (2.7%)	1 (3.1%)	1 (2.3%)	
**Tumor site**				
Bilateral	1 (1.3%)	0 (0%)	1 (2.3%)	0.662
Left kidney	36 (48.0%)	15 (46.9%)	21 (48.8%)	
Right kidney	38 (50.7%)	17 (53.1%)	21 (48.8%)	
**Distant metastasis**				
No	73 (97.3%)	31 (96.9%)	42 (97.7%)	1
Yes	2 (2.7%)	1 (3.1%)	1 (2.3%)	

### CORO6 Enhancement of ccRCC Cell Growth

**Figure 5:**
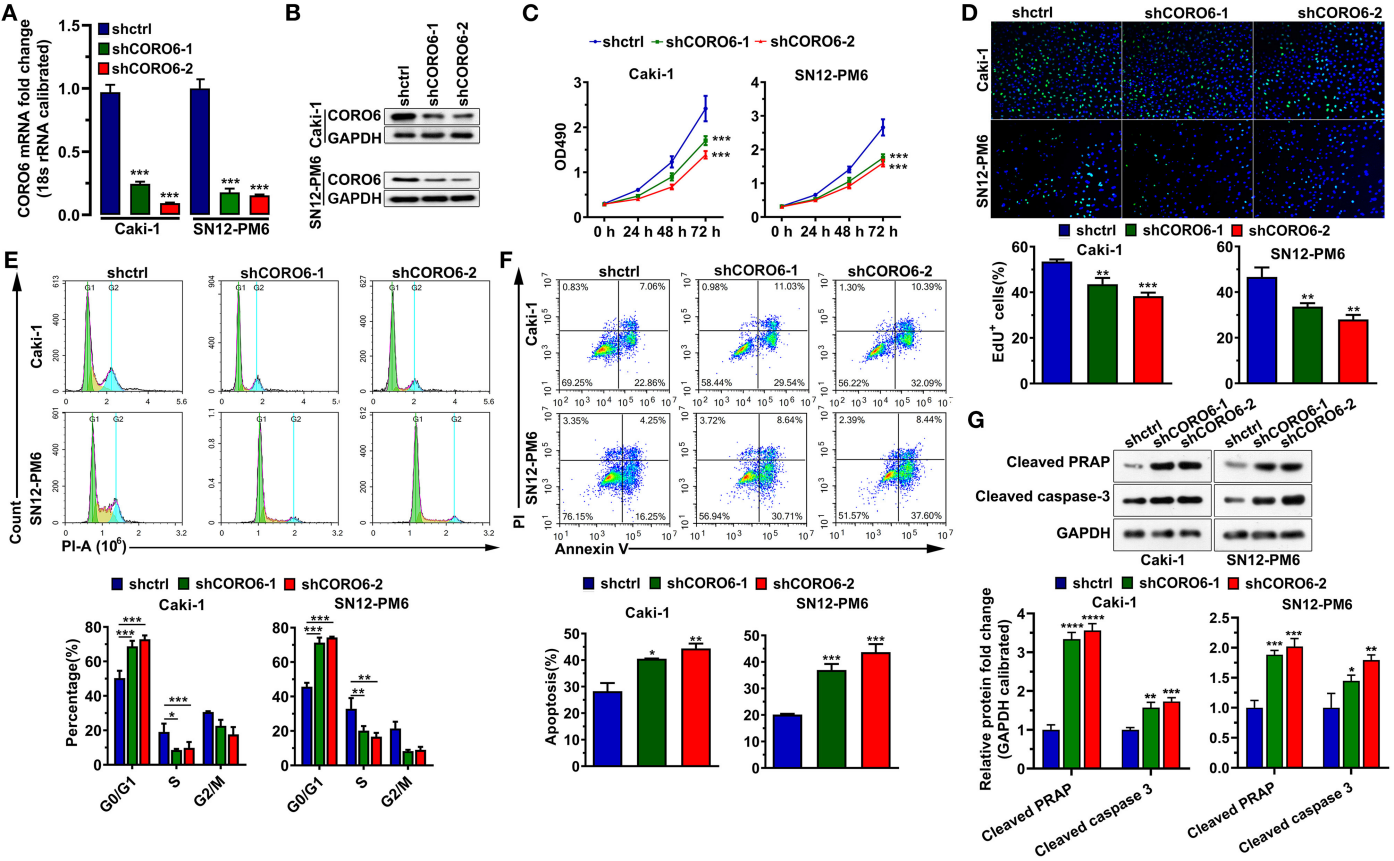
CORO6 enhanced ccRCC cell growth. **(A,B)** Knockdown efficiency of shRNAs against CORO6 by qPCR **(A)** and Western blotting **(B)**. **(C)** The MTT assay revealed that CORO6 reduction suppressed Caki-1 and SN12-PM6 cell growth. **(D)** EdU staining showed that CORO6 reduction decreased Caki-1 and SN12-PM6 cell proliferation. Top: representative images of EdU staining. Bottom: statistical analyses. **(E)** PI-stained flow cytometry analysis showed that CORO6 depletion caused cell cycle arrest at G0/G1 in Caki-1 and SN12-PM6 cells. **(F)** Annexin V-/PI-stained flow cytometry analysis indicated that CORO6 depletion increased Caki-1 and SN12-PM6 cell apoptosis. **(G)** Western blotting analyses revealed that CORO6 knockdown increased expression levels of cleaved PRAP and cleaved caspase-3. Top, representative image of western blotting analyses. Bottom, a statistical analyses. GAPDH was a loading control. *P < 0.05, **P < 0.01, ***P < 0.001, and ****P < 0.0001.

To test whether CORO6 contributed to ccRCC development, we first knocked down CORO6 by two independent short hairpin RNAs (shRNAs) in Caki-1 and SN12-PM6 cells. Our results showed that these two shRNAs against CORO6 successfully depleted CORO6 at both the mRNA and protein levels (Figures 5A,B). As expected, the abrogation of CORO6 significantly reduced Caki-1 and SN12-PM6 cell growth, which was monitored by an MTT assay (Figure 5C). Cell growth is at least somewhat determined by cell proliferation and cell apoptosis. To further identify the roles of CORO6, we performed EdU staining to examine ccRCC cell proliferation with or without CORO6 manipulation. The results illustrated that the numbers of EdU-positive Caki-1 and SN12-PM6 cells were considerably decreased when CORO6 was depleted (Figure 5D), indicating that CORO6 attenuation may suppress Caki-1 and SN12-PM6 cell proliferation. In addition, we also found that the loss of CORO6 in Caki-1 and SN12-PM6 cells led to cell cycle arrest at the G0/G1 phase (Figure 5E) and annexin V/PI-monitored cell apoptosis (Figure 5F). Western blotting detections of apoptotic markers cleaved PRAP and cleaved caspase-3 also confirmed that knockdown of CORO6 led to cell apoptosis of Caki-1 and SN12-PM6 cells (Figure 5G). Together, all of these data suggest that CORO6 promotes ccRCC cell growth by increasing cell proliferation and suppressing cell apoptosis.

### CORO6 Promotion of ccRCC Cell Migration and Invasion

**Figure 6:**
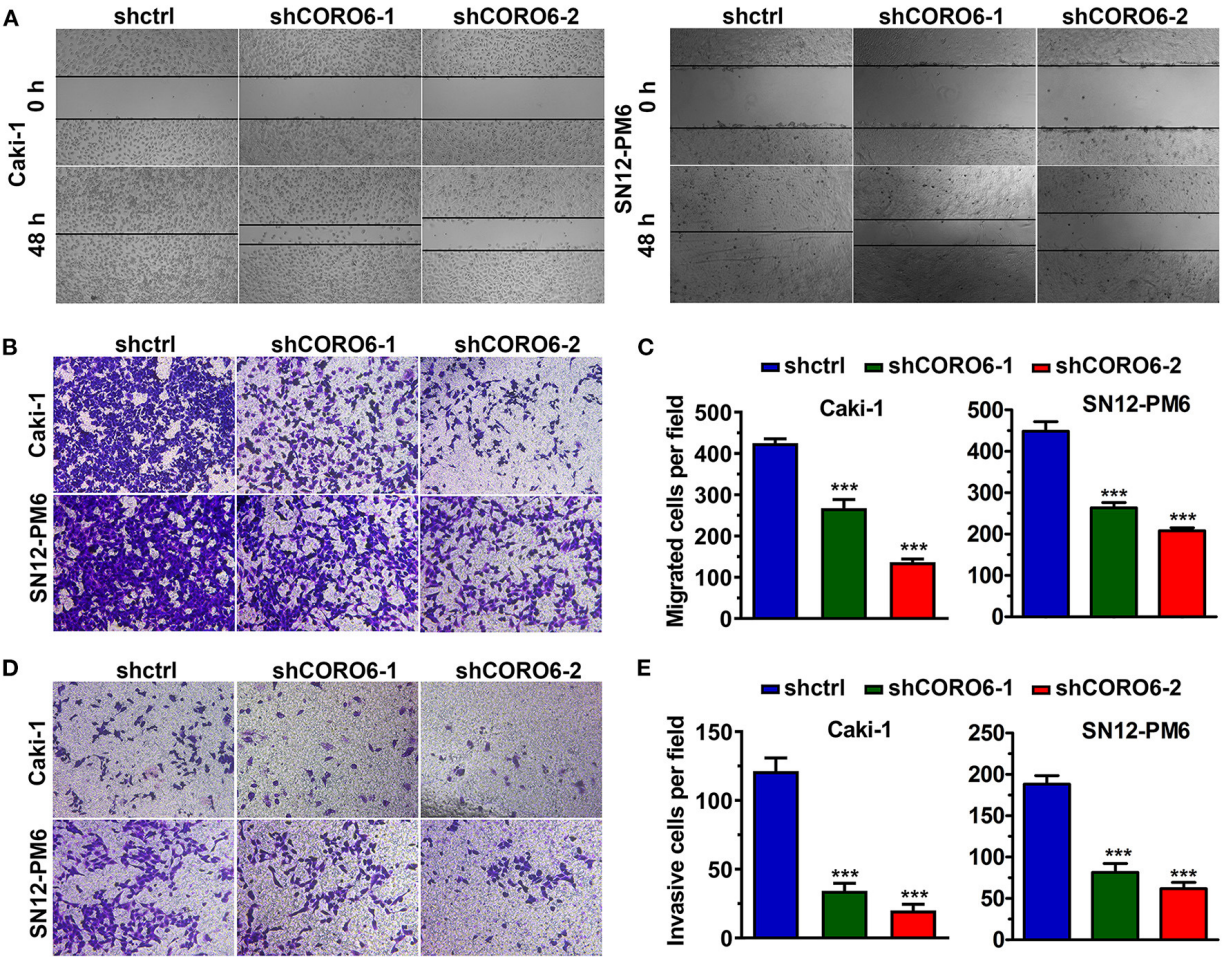
CORO6 promoted ccRCC cell migration and invasion. **(A)** The wound healing assay showed that knockdown of CORO6 inhibited Caki-1 and SN12-PM6 cell migration. **(B,C)** The transwell migration assay revealed that CORO6 knockdown suppressed Caki-1 and SN12-PM6 cell migration. **(B)** Representative images of migrating cells. **(C)** Statistical analysis of **(B)**. **(D,E)** CORO6 knockdown significantly suppressed the invasion ability of Caki-1 and SN12-PM6 cells. **(D)** Representative images of invading cells. **(E)** Statistical analysis of **(D)**. ***P < 0.001.

Cell migration and cell invasion are two essential hallmarks of cancer cells (Hanahan and Weinberg, 2011). Given this notion, we sought to test whether CORO6 contributes to cell migration and cell invasion of ccRCC cells. The wound healing assay illustrated that a strong inhibition of Caki-1 and SN12-PM6 cell migration was clearly observed when CORO6 expression was efficiently attenuated (Figure 6A). To confirm this, we performed a transwell migration assay, which revealed that CORO6 knockdown was indeed able to suppress ccRCC cell migration (Figures 6B,C). In addition, the results from the Matrigel invasion assay also showed that loss of CORO6 in Caki-1 and SN12-PM6 cells dramatically inhibited their invasion ability (Figures 6D,E). In summary, all of these findings indicate that CORO6 promotes ccRCC cell migration and invasion.

### Mechanistic Dissection of CORO6 Promotion of ccRCC Progression

**Figure 7:**
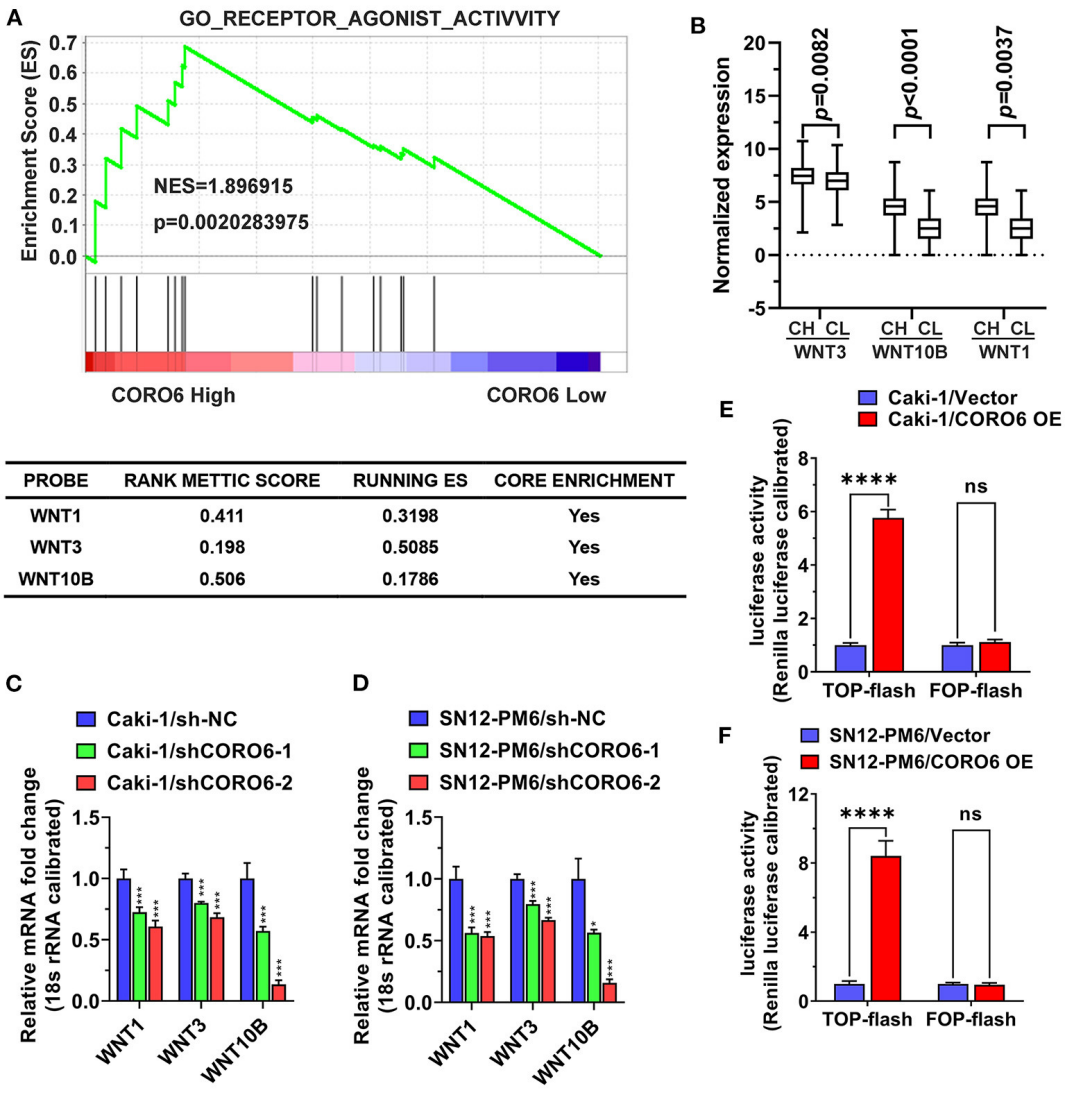
Mechanistic dissection of how CORO6 promoted ccRCC progression. **(A)** GSEA KEGG analyses showed that GO receptor agonist activity was highly enriched in the high CORO6 group. **(B)** The expression levels of WNT1, WNT3, and WNT10B increased in ccRCC patients with a high level of CORO6. **(C,D)** CORO6 knockdown dramatically reduced the expression levels of WNT1, WNT3, and WNT10B in both Caki-1 **(C)** and SN12-PM6 cells **(D)**. The expression levels of detected genes were normalized to 18s rRNA. **(E,F)** CORO6 enhanced the activity of TOPflash but not FOPflash in both Caki-1 **(E)** and SN12-PM6 cells **(F)**. *P < 0.05, ***P < 0.001, and ****P < 0.0001. Ns, no significance.

To identify the underlying mechanisms responsible for CORO6-mediated ccRCC cell growth and cell migration/invasion, we first grouped patients from the TCGA KIRC dataset into high and low CORO6 groups using the median expression level of CORO6 as a cutoff and performed KEGG pathway analyses using the GSEA tool. The data revealed that Gene Ontology (GO) receptor agonist activity was tightly associated with the CORO6 expression level (Figure 7A). Subsequent analysis pinpointed that WNT1, WNT3, and WNT10B were highly enriched in the high CORO6 group (Figure 7B), indicating that CORO6 may regulate the WNT pathway to affect ccRCC cell growth and invasion. Indeed, knockdown of CORO6 reduced the expression levels of WNT1, WNT3, and WNT10B in Caki-1 and SN12-PM6 cells (Figures 7C,D). In addition, TOPflash luciferase activity, which is used to monitor the WNT signaling pathway, was clearly stimulated by CORO6 in Caki-1 and SN12-PM6 cells. However, CORO6 failed to activate negative FOPflash activity (Figures 7E,F). Taken together, these results prove that WNT signaling is one of the mechanisms responsible for CORO6-mediated ccRCC development.

### CORO6-Induced ccRCC Cell Growth and Cell Invasion/Migration Associations With WNT Activation

**Figure 8:**
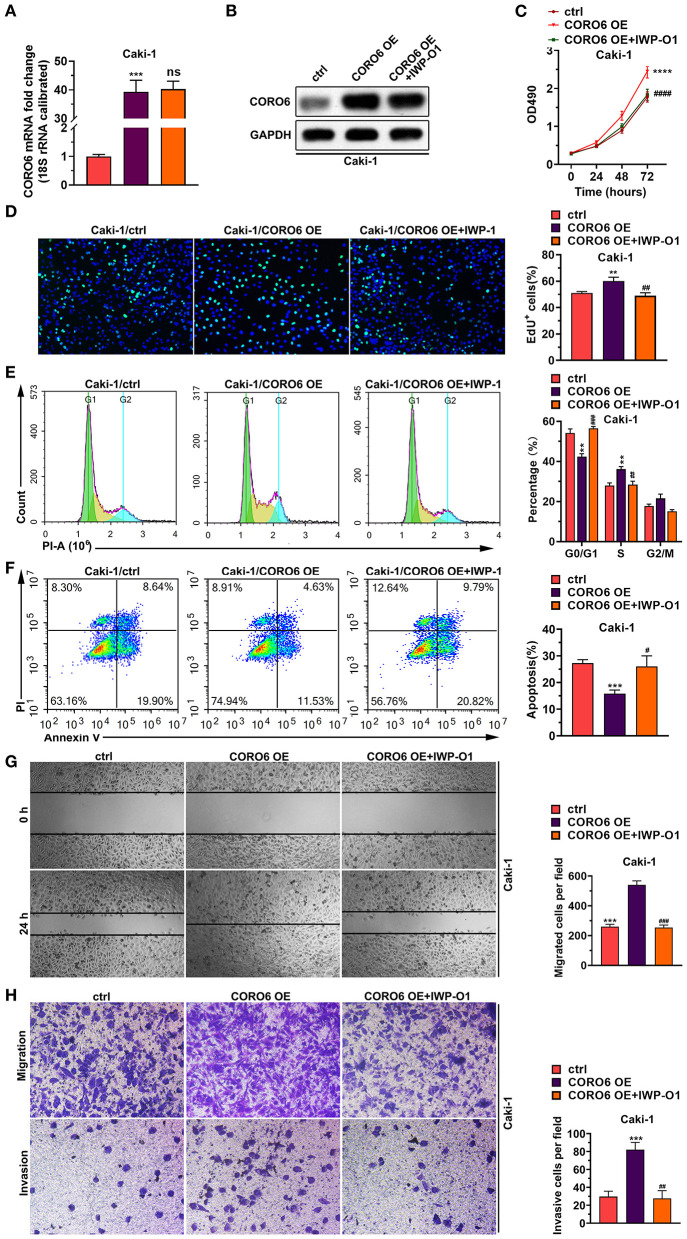
CORO6-induced ccRCC cell growth and cell invasion/migration were dependent on WNT activation. **(A)** IWP-O1 had no effect on the CORO6 mRNA level. 18s rRNA was used as the normalizing control. **(B)** IWP-O1 had no effect on the CORO6 protein level. GAPDH served as the loading control. **(C)** The MTT assay showed that IWP-O1 blocked CORO6-induced cell growth of Caki-1 cells. **(D)** EdU staining revealed that IWP-O1 inhibited CORO6-increased cell proliferation of Caki-1 cells. Left: representative image of EdU staining. Right: statistical analysis. **(E)** PI-stained flow cytometry analysis showed that CORO6-mediated cell cycle progression from G0/G1 to the S phase of Caki-1 cells was blocked by IWP-O1. Left: representative image of PI-stained flow cytometry analysis. Right: statistical analysis. **(F)** Annexin V-/PI-stained flow cytometry analysis indicated that CORO6-decreased cell apoptosis of Caki-1 cells was reversed by IWP-O1 treatment. Left: representative image of annexin V-/PI-stained flow cytometry analysis. Right: statistical analysis. **(G)** CORO6-induced Caki-1 cell migration was suppressed by IWP-O1 treatment. Left: representative images of migrating cells. Right: statistical analysis of migrating cells. **(H)** CORO6-induced Caki-1 cell invasion was suppressed by IWP-O1 treatment. Left: representative images of invading cells. Right: statistical analysis. **P < 0.01, ***P < 0.001, and ****P < 0.0001. Compared with CORO6 OE, ^#^*P* < 0.05, ^*##*^*P* < 0.01, ^*###*^*P* < 0.001, and ^*####*^*P* < 0.0001.

To validate the involvement of WNT signaling in CORO6-induced ccRCC cell growth and cell invasion/migration, we sought to explore whether WNT signaling inhibitor IWP-O1 could attenuate the CORO6 effect on ccRCC cells. Of note, our data illustrated that IWP-O1 had no ability to affect the expression level of CORO6 at both the mRNA and protein levels (Figures 8A,B), suggesting that the anti-cancer effect of IWP-O1 was due to WNT inhibition but not the indirect effect from CORO6 reduction. As expected, CORO6-induced Caki-1 cell growth was reversed in the presence of IWP-O1 (Figure 8C). Inhibition of WNT signaling also prevented CORO6 from increasing the cell proliferation rate (Figure 8D), turning cell cycle entry into the S phase (Figure 8E) and decreasing Caki-1 cell apoptosis (Figure 8F). All of these data suggest that CORO6-induced cell growth of ccRCC cells was at least partially caused by WNT activation.

In addition, we also found that CORO6 lost its ability to increase Caki-1 cell migration (Figure 8G) and cell invasion (Figure 8H) when there cells were supplemented with IWP-O1, suggesting that the activation of WNT signaling contributes to CORO6-induced cell invasion/migration.

Taken together, the data from Figures 8A–H suggest that the phenotypic alterations of ccRCC cells caused by CORO6 were partially attributable to WNT activation.

### Xenograft Mouse Model to Support the Oncogenic Role of CORO6 in ccRCC Development

**Figure 9:**
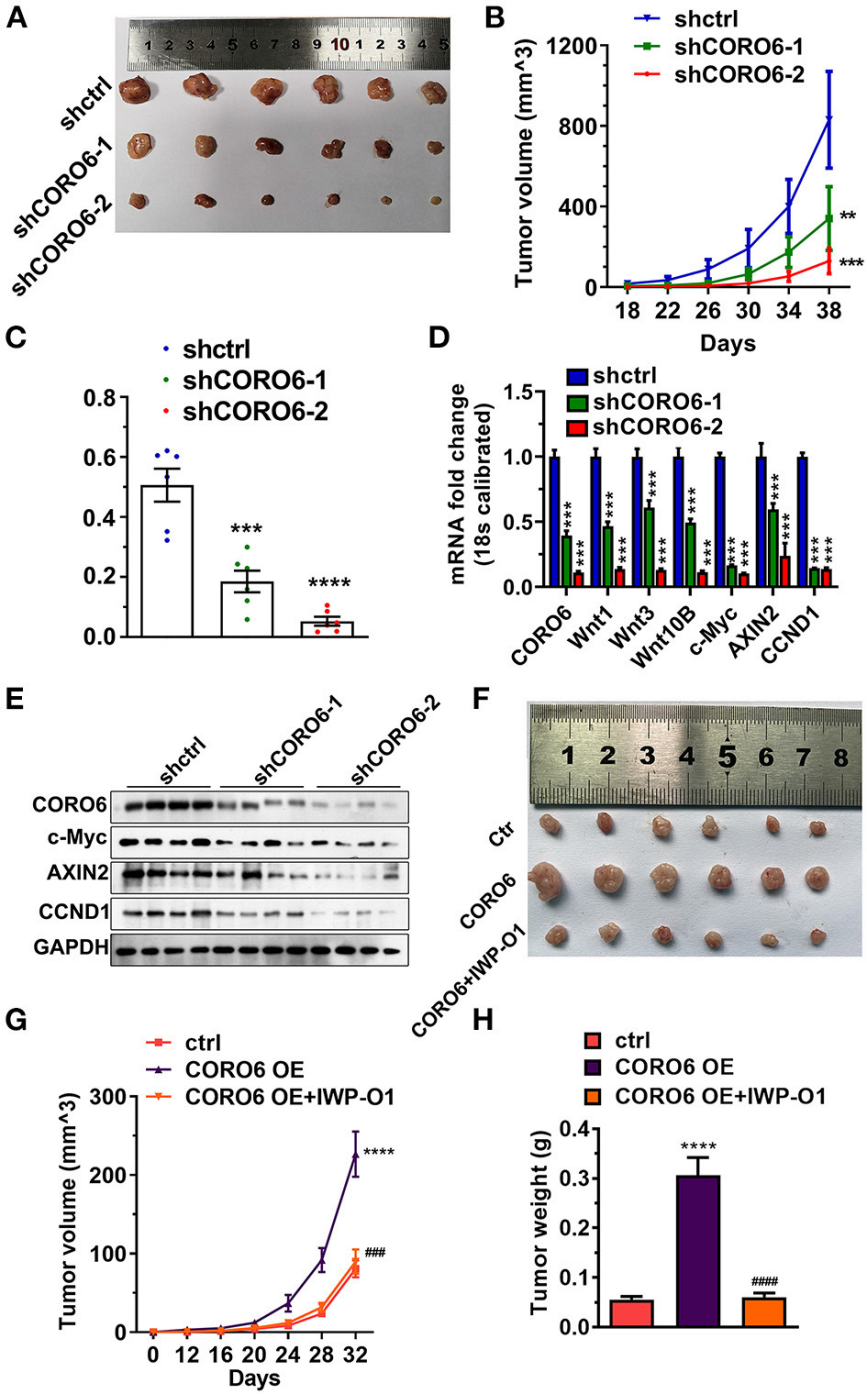
Xenograft mouse model to support the oncogenic role of CORO6 in ccRCC development. **(A–C)** CORO6 depletion suppressed ccRCC tumor growth monitored by tumor size **(A)**, growth curve **(B)**, and tumor weight **(C)**. **(D)** The mRNA levels of c-Myc, AXIN2, and CCND1 were remarkably reduced in CORO6-depleted ccRCC tumors. The expression levels of detected genes were normalized to 18s rRNA. **(E)** Western blotting showed that the protein levels of c-Myc, AXIN2, and CCND1 were remarkably reduced in CORO6-depleted ccRCC tumors. GAPDH was used as the internal control. **(F–H)** CORO6-promoted ccRCC tumor growth was inhibited by the administration of IWP-O1 monitored by tumor size **(F)**, growth curve **(G)**, and tumor weight **(H)**. **P < 0.01, ***P < 0.001, and ****P < 0.0001. Compared with CORO6 OE, ^*###*^*p* < 0.001 and ^*####*^*p* < 0.0001.

To translate our findings into a pre-clinical animal model, we first used lentivirus system to generate shCtrl- or shCORO6- Caki-1 cells, then subcutaneously implanted 1 ×10^6^ cells within 200 μl Matrigel into nude mice and monitored ccRCC tumor growth. Consistent with our *in vitro* findings, CORO6 depletion by two independent shRNAs led to a significant suppression of ccRCC tumor growth monitored by tumor size (Figure 9A), growth curve (Figure 9B), and tumor weight (Figure 9C), suggesting that CORO6 indeed acted as a tumor-promoting factor in the development of ccRCC. Importantly, our data also showed that CORO6-depleted ccRCC tumors expressed low mRNA expression levels of WNT1, WNT3, and WNT10B (Figure 9D). Moreover, protein detections of the well-known downstream targets of WNT signaling, including c-Myc, AXIN2, and CCND1, showed that they were dramatically reduced in CORO6-depleted tumors (Figure 9E). Furthermore, a second xenograft mouse model validated that CORO6 significantly promoted ccRCC tumor growth (Figures 9F–H) attenuated by WNT signaling inhibitor IWP-O1 (Figures 9F–H), suggesting the involvement of WNT signaling in CORO6-mediated ccRCC growth *in vivo*.

## Discussion

Identification of novel therapeutic targets for ccRCC remains a primary scientific research focus. In this study, we found that the CORO6 level was significantly increased in ccRCC patients compared to normal kidney tissues. Importantly, the CORO6 level was tightly associated with tumor metastasis and OS of ccRCC patients, strengthening the prognostic value of CORO6 in ccRCC patients. Our experimental findings also suggest that CORO6 functions to promote cell growth and cell migration/invasion of ccRCC cells, which may be attributable to the activation of WNT signaling. Moreover, the results from an *in vivo* xenograft mouse model revealed that CORO6-depleted ccRCC tumors grew more much slowly than control ones. Overall, our study defines the oncogenic role of CORO6 in ccRCC development and provides a rationale for developing CORO6 targeted-therapies for improved treatment of ccRCC patients.

Coronin family members have recently been recognized for their role in cancer development. We previously found that CORO3 serves as tumor-promoting factor to control cell growth and invasion of ccRCC cells. Downregulation of CORO3 by its specific upstream miRNA, miR-26, appeared to prevent CORO3-induced cell growth and invasion (Wang et al., 2020). Other studies have also consistently documented that CORO proteins are overexpressed in various cancers, including breast cancer, gastric cancer, and hepatocellular carcinoma (Thal et al., 2008; Wu et al., 2010). For gastric cancer in particular, CORO3 has been observed to promote cancer metastasis by upregulating the expression levels of MMP9 and cathepsin K (Ren et al., 2012). All of these data suggest that CORO proteins play an oncogenic role in cancer carcinogenesis. Here, we investigated CORO6 as a tumor-promoting factor in ccRCC development. As actin-binding proteins, coronin family members may affect the dynamics of actin polymerization, which in turn influences the protrusive structures of cancer cells with invading or migrating potential. Therefore, disruption in the CORO6 level may prevent the formation of protrusive structures and subsequently impair the invading capacity of cancer cells.

Moreover, we observed CORO6 regulation of WNT signaling by increasing WNT1, WNT3, and WNT10B, suggesting that CORO6-induced ccRCC cell growth and invasion may be attributable to the activation of WNT signaling. Of note, WNT signaling in ccRCC development has been widely recognized. Previous documents have shown that high expression levels of WNT1 and WNT10B were observed in ccRCC tissues and cell lines (Hsu et al., 2012; Kruck et al., 2013; Xu et al., 2016). Furthermore, both WNT1 and WNT10B were independent predictors for prognosis in ccRCC patients. In addition, most other molecules involved in WNT signaling were also dysregulated in ccRCC patients, highlighting the pivotal role of WNT signaling in the progression of this type of cancer. Indeed, inhibitors specific to WNT signaling are being widely developed (Koller et al., 2013; VON Schulz-Hausmann et al., 2014) and they may present promising therapeutic applications in the treatment of ccRCC patients.

The mechanism by which CORO6 activates WNT signaling remains unknown and deserves further investigation. We hypothesize that with the WD40 repeat domain, CORO6 may serve as a scaffold protein to regulate numerous signaling pathways, including WNT signaling. It is possible that CORO6 interacts with certain proteins that serve as essential components of WNT signaling to control RCC progression. In summary, our study identifies CORO6 as either a novel potential biomarker or therapeutic target for improved treatment of ccRCC patients.

## Data Availability Statement

The datasets presented in this study can be found in online repositories. The names of the repository/repositories and accession number(s) can be found in the article/supplementary material.

## Ethics Statement

The studies involving human participants were reviewed and approved by Institutional Review Board of Xiamen University. The patients/participants provided their written informed consent to participate in this study. The animal study was reviewed and approved by Institutional Review Board of Xiamen University.

## Author Contributions

GCL oversaw the study. XJW, YMX, and SL performed the experiments and wrote manuscript. ZJY analyzed data. All authors contributed to the article and approved the submitted version.

## Conflict of Interest

The authors declare that the research was conducted in the absence of any commercial or financial relationships that could be construed as a potential conflict of interest.
